# Amenability of South African Banded Iron Formation (BIF) to Fines Gravity Processing

**DOI:** 10.1007/s42461-023-00758-6

**Published:** 2023-05-12

**Authors:** Carla Da Corte, Ashma Singh, Kagisego Letsoalo

**Affiliations:** Mintek, Johannesburg, Gauteng South Africa

**Keywords:** South Africa, Banded Iron Formation, Gravity separation, Amenability

## Abstract

Banded Iron Formations (BIF) are sedimentary rock formations ranging in age from 0.8 to 3.8 billion years and consist of alternating layers of silica and iron. The thickness of the alternating layers varies between and within deposits, with this lithology forming approximately two-thirds of South Africa’s future low-grade hematite resources. The production costs for South African iron ore producers are approximately double that of the largest iron ore producers, namely, Brazil and Australia. This in conjunction with volatile commodity prices, necessitated a cost-sensitive beneficiation strategy for low-grade hematite to sustain the industry and extend life of mine. A BIF sample grading at 44% Fe and comprising predominantly of hematite and quartz with minor amounts of magnetite and goethite was subjected to three fines gravity processing routes to establish the amenability of this sample to beneficiation. To provide flexibility for iron ore producers who still have high-grade resources available, two product grades were considered, namely a 60% Fe product for further blending or a 63% Fe product for direct sales.

## Introduction

Banded Iron Formations (BIF) are sedimentary rock formations with alternating silica-rich layers and iron-rich layers that are typically composed of iron oxides (hematite and magnetite), iron-rich carbonates (siderite and ankerite) and/or iron-rich silicates (e.g. minnesotaite and greenalite). These formations range in thickness from several to several hundred metres, and extend from a few to several hundred kilometres [1].

BIF has been observed in rocks as old as 3.8 billion years from Southwest Greenland and as young as 0.8 billion years in northwest Canada [1]. Klein and Beukes (1992 as cited by [2] reveal that the Hamersley Basin in Western Australia which is 2.45 billion years old is the single largest known BIF deposit worldwide.

**Fig. 1:**
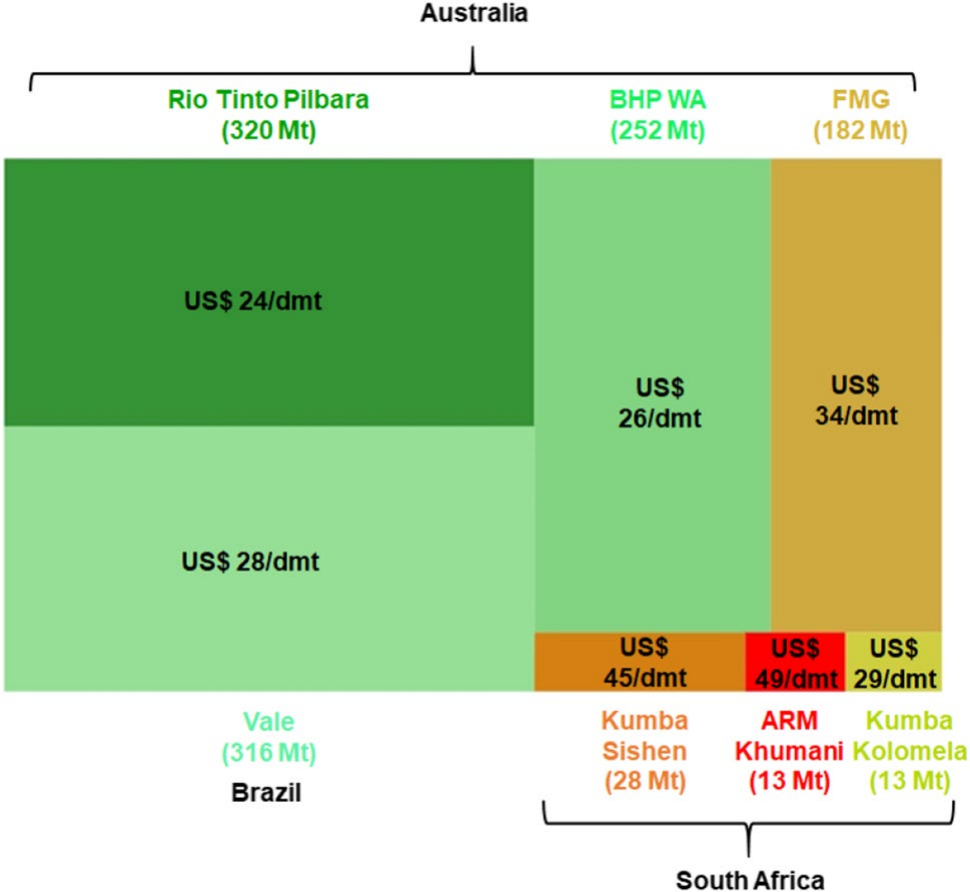
Global seaborne iron ore costs per producer as function of 2021 production volumes (adapted from: [3–7,8, 9]

Within the next 8 years, South Africa’s high-grade iron ore resources will be depleted and exploration of low-grade resources (< 50–55% Fe) is becoming more prominent to sustain the industry and extend life of mine. BIF is expected to constitute approximately two-thirds of the low-grade material and thus it is important to understand its beneficiation potential. However, the volatile commodity prices and the high production cost for South African producers, approximately 1.5 times that of largest iron ore–producing countries like Australia and Brazil as shown in Fig. 1, will need to be considered when developing a beneficiation strategy for South African BIF. The size of the blocks in Fig. 1 represents the relative production volumes achieved by each producer in 2021 along with the cost for iron ore production in US dollars per dry metric ton, with the lowest to highest cost producers ranging in colour from green to red respectively.

In light of these challenges and the increasing input costs associated with mining, electricity and COVID-19, a fines gravity separation process was investigated to determine the amenability of the BIF sample to generate products grading at 60–63% Fe. The process encompassed milling and shaking table test work via three circuit configurations.

## Experimental

### Sample Preparation and Characterisation

A BIF hematite sample originating from the Limpopo province of South Africa was utilised for the study.

A 150 kg aliquot of the as-received sample was stage crushed to − 1.18 mm utilising jaw and cone crushers and subsequently blended and sub-sampled for head analysis, size-by-assay, mineralogy and gravity tests. Table 1 indicates that the BIF sample grades at 44% Fe with silica as the dominant contaminant species.

**Table 1:** Head assay

Fe	Si	Al	Mg	Mn	Ca	Ti	LOI
%	%	%	%	%	%	%	%
44.00	16.12	0.50	0.07	0.20	0.05	0.10	0.48

The discrete mass, Fe grade and deportment for the crusher product indicate a bimodal mass distribution with peaks in the − 850 + 600 µm and − 38 µm size fractions, with 56.4% + 212 µm. The Fe deportment closely follows that of mass indicating that the Fe grade remains relatively constant across size.

### Mineralogy on the − 1.18 mm Material

#### Electron Microprobe Analysis

A polished section of the − 1.18 mm crushed material was analysed on the Cameca SX 50 electron microprobe using wavelength dispersive spectrometry. An accelerating voltage of 20 kV was used with a beam current of 30 nA. The system was calibrated using pure oxide reference standards and were cross-referenced to the hematite standards during measurement to ensure reproducibility and quality of results. Oxygen content was calculated by stoichiometry.

#### Particle Tracking Analysis

Three size fractions, namely + 850 µm, − 850 + 212 µm and -212 µm, were each prepared into sections, carbon coated and analysed using autoSEM (Mineral Liberation Analyser [MLA]). One to two sections were prepared for each size fraction for statistical confidence.

During measurement, the MLA generates an X-ray analysis for each region (grey level) within a particle. The measurement mode employed in this study was chosen on the basis of the ore type, run time, purpose of study, particle size and the successful ability to delineate mineral grain boundaries in particles. During the investigation, between 2500 and 20,000 particles per polished section for each fraction were analysed and processed via AutoSEM. A larger number of particles aid in the acquisition of a statistically representative dataset of the overall sample.

The data for each size fraction was mathematically combined into the correct ratio to represent the overall sample. Particle characterisation data pertaining to mineral types/compositions, particle size, density, weight percent of the particle population, area of particle, shape factor, circularity and perimeter of each particle were ascertained during offline processing. The data were used to characterise the sample in terms of mineral, density and size distribution of the sample. The liberation characteristics of each mineral by size class were also extracted.

### Shaking Table Test Work

Three 5 kg aliquots were screened at 212 µm using a Rhologan; and the screened oversize and undersize were prepared accordingly as required by each flowsheet option. Table 2 shows operating conditions for all of the shaking tables in the three flowsheet options.

**Table 2:** Operating conditions of the shaking tables

Operating conditions	Option 1 and option 2	Option 1 and option 2	Option 2	Option 3
	+ 212 µm	− 212 µm	+ 212 µm ST concentrate	Prepared ST feed
Water in funnel [L/hr]	200	200	200	200
Water in mixing box [L/hr]	200	200	200	200
Spray water (table) [L/hr]	300	300	300	300
Table angle [°]	29	23	23	20
Solids feed rate [kg/hr]	25.9	25.2	25.9	27.8
Solids feed rate [g/min]	432	420	432	464

In all options, a 1/8 Wilfley shaking table was fed with the relevant size fraction. Each shaking table test produce 9 products, that is 4 concentrates, 2 middlings, 2 tailings and 1 slimes products.

**Fig. 2:**
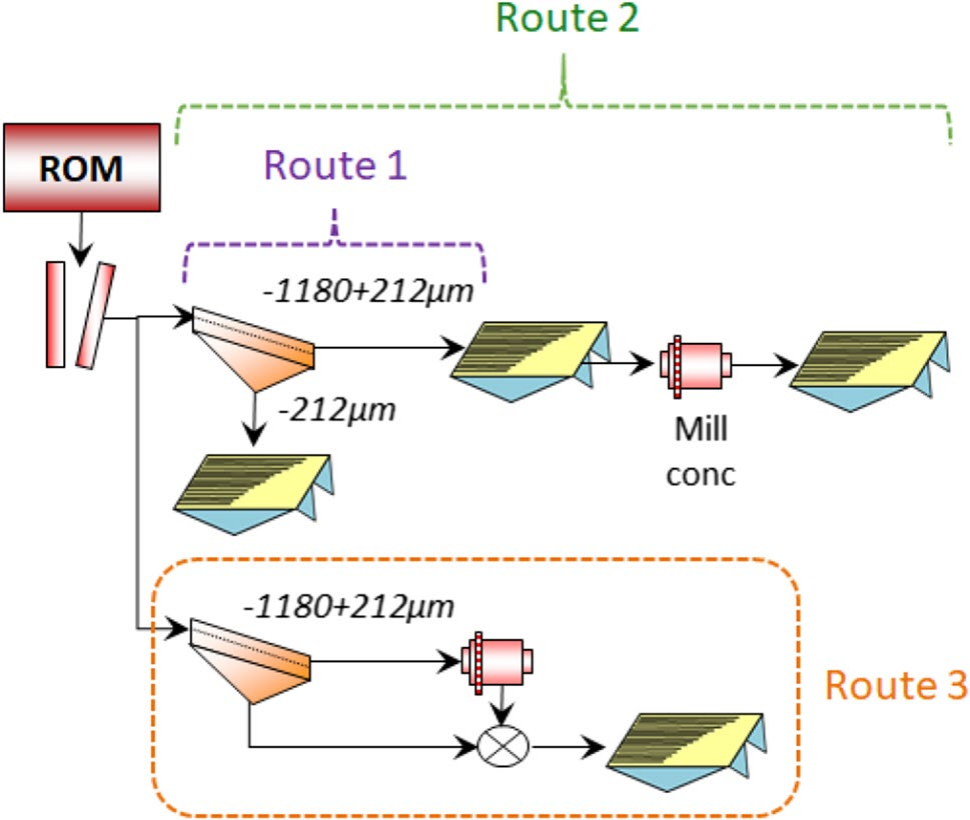
The three gravity processing routes considered

Three circuits considered in this study are presented in Fig. 2.

The first option, the base case, includes screening the material at 212 µm and processing the undersize and oversize independently across the shaking table. The second option is similar to the first except the rougher concentrate from the + 212 µm size fraction is further milled to ~ 80% passing 212 µm to improve the liberation characteristics and subjected to one final cleaning stage. In the final option, the screen oversize is milled to ~ 80% − 212 µm and composited with the natural − 212 µm prior to shaking table test work. The − 212 µm shaking table bulk concentrate (route 2) and the + 212 µm size fraction (route 3) were wet milled at various times and a milling curve constructed to determine the milling time required to achieve a grind of ~ P80 212 µm. The milling conditions and ball charge are presented in Table 3 and Table 4 respectively.

**Table 3:** Ball mill operating conditions

Liners:	Rubber lined
Lifters:	3 lifter bars
Diameter (cm):	20
Length (cm):	25
Speed (rpm):	72
Mass of solids (kg)	1
Volume of water (mL)	430
Mass of water (kg)	0.43
% Solids	70
Milling targets	P80 212 μm

**Table 4:** Ball mill charge

Ball size (mm)	Mass (kg)	No of balls	Mass (%)	Cumulative mass%
35	6.57	52	41.9	41.9
30	2.97	42	18.9	60.8
25	5.54	131	35.3	96.2
20	0.6	25	3.8	100
Total	15.68		100	

The mill discharge was blended and sub-sampled for sizing and either composited with the natural − 212 µm fraction or subjected to shaking table test work directly. The resulting grind of the products is reported in Table 5.

**Table 5:** Grinds at the milling exit

Approximate grinds after milling	P30	P50	p80
Route 2 PSD	40.0	84.0	212.0
Route 2 PSD	50.0	120.0	212.0
Composite feed (route3)	28.0	80.0	167.0

## Results and Discussion

### Mineralogy on the − 1.18 mm Material

#### Electron Microprobe Analysis

EMPA was conducted on the − 1.18 mm feed in order to classify the hematite phases correctly and then reliably assign Fe grade and density to each phase identified. The point analysis indicates the average Fe content for hematite is 69.23% as shown in Table 6. A mineral reference list is used to distinguish mineral phases.

**Table 6:** EMPA determined chemistry by point analysis (5 µm spot size)

*n* = 92	Fe	Si	Al	Mg	Mn	Ca	Ti	K
MIN	67.87	-	-	-	-	-	-	-
MAX	70.14	0.73	0.23	0.2	0.21	0.14	0.37	0.03
AVG	69.23	0.17	0.09	0.07	0.05	0.04	0.05	0.02

Based on the EMPA data, it was observed that the Fe grade for hematite varied substantially when the raster scan over an area of 100 µm × 75 µm was used. This was primarily due to the intergrown nature of the hematite and quartz minerals. As a result, it was decided it would be best to classify the different hematite phases present in the sample. Table 7 presents three different hematite phases each with its own density and chemistry. Hematite phase A is the purest hematite form with Fe grades of 69.44% whilst the B and C phases grade at 67.22% Fe and 62.44% Fe respectively.

**Table 7:** Chemistry of the three defined hematite phases

Mineral	Density (g/cm^3^)	Fe (%)	Si (%)	Al (%)	Mg (%)	Mn (%)	Ca (%)	K (%)	O (%)
Hematite phase A	5.15	69.44	0.33	0.33	0.00	0.00	0.07	0.06	30.34
Hematite phase B	5.12	67.22	0.53	0.53	0.00	0.00	0.00	0.00	29.9
Hematite phase C	4.85	62.44	4.65	0.18	0.04	0.07	0.04	0.07	28.88

#### Particle Tracking Analysis

Table 8 shows the mineral abundance and indicates hematite phase C is the mineral present with minor proportions of the hematite phase B and magnetite. The dominant gangue is quartz which correlates with the low contaminant levels observed in the head assay. Considering the two hematite phases in the sample, their mineral chemistry and the proportion of each in the sample, a product grading between 62.44 and 67.22% Fe, could be achieved for the − 1180 µm feed.

**Table 8:** Mineral abundance of the − 1180 µm laboratory cone crusher product

Mineral	Density (g/cm^3^)	Mass (%)
Hematite phase A	5.15	0.0
Hematite phase B	5.12	3.2
Hematite phase C	4.85	72.1
Quartz	2.65	23.2
Magnetite	5.15	1.5
Goethite	3.8	0.0
Calcite	2.71	0.0
Dolomite	2.85	0.0
Total	4.07	100.0

**Fig. 3:**
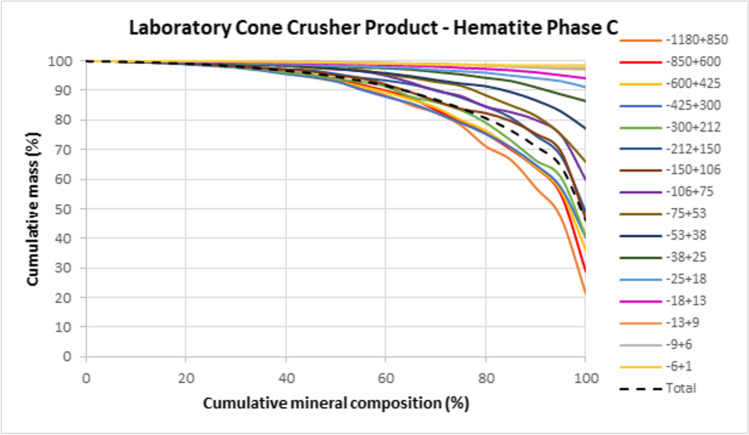
Hematite phase C liberation characteristics for the − 1180 µm laboratory cone crusher product

The liberation by area characteristics of the dominant hematite phase by size is presented in Fig. 3 and demonstrates that approximately 80% of hematite phase C is liberated in the 80% liberated class with the + 212 µm size fraction being less liberated than the total. Table 9 demonstrates that for ~ 80% of the hematite phase C to be 80%, 90% and 100% liberated classes then grind sizes of 212 µm; 106 µm and 38 µm would be required respectively. As a result, a grind of 212 µm was selected for the three gravity routes defined earlier.

**Table 9:**
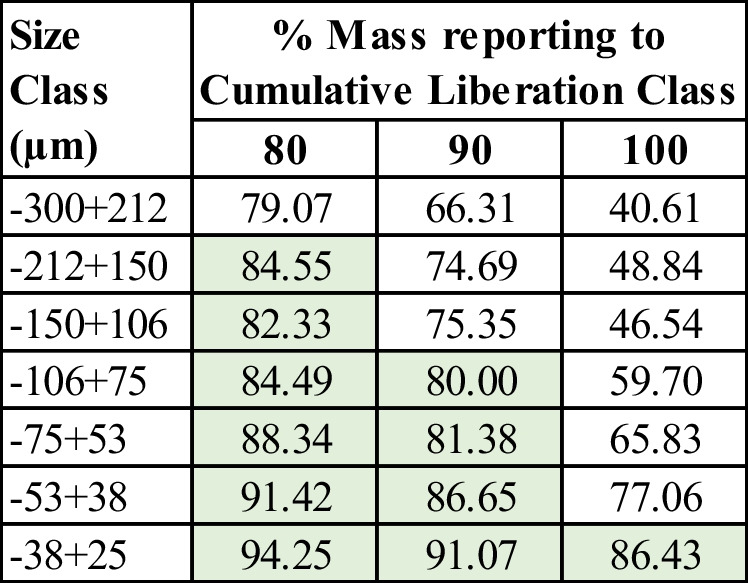
Hematite phase C liberation by size of the − 1180 µm laboratory cone crusher product.

Indication of the approximate P80 per liberation class

**Fig. 4:**
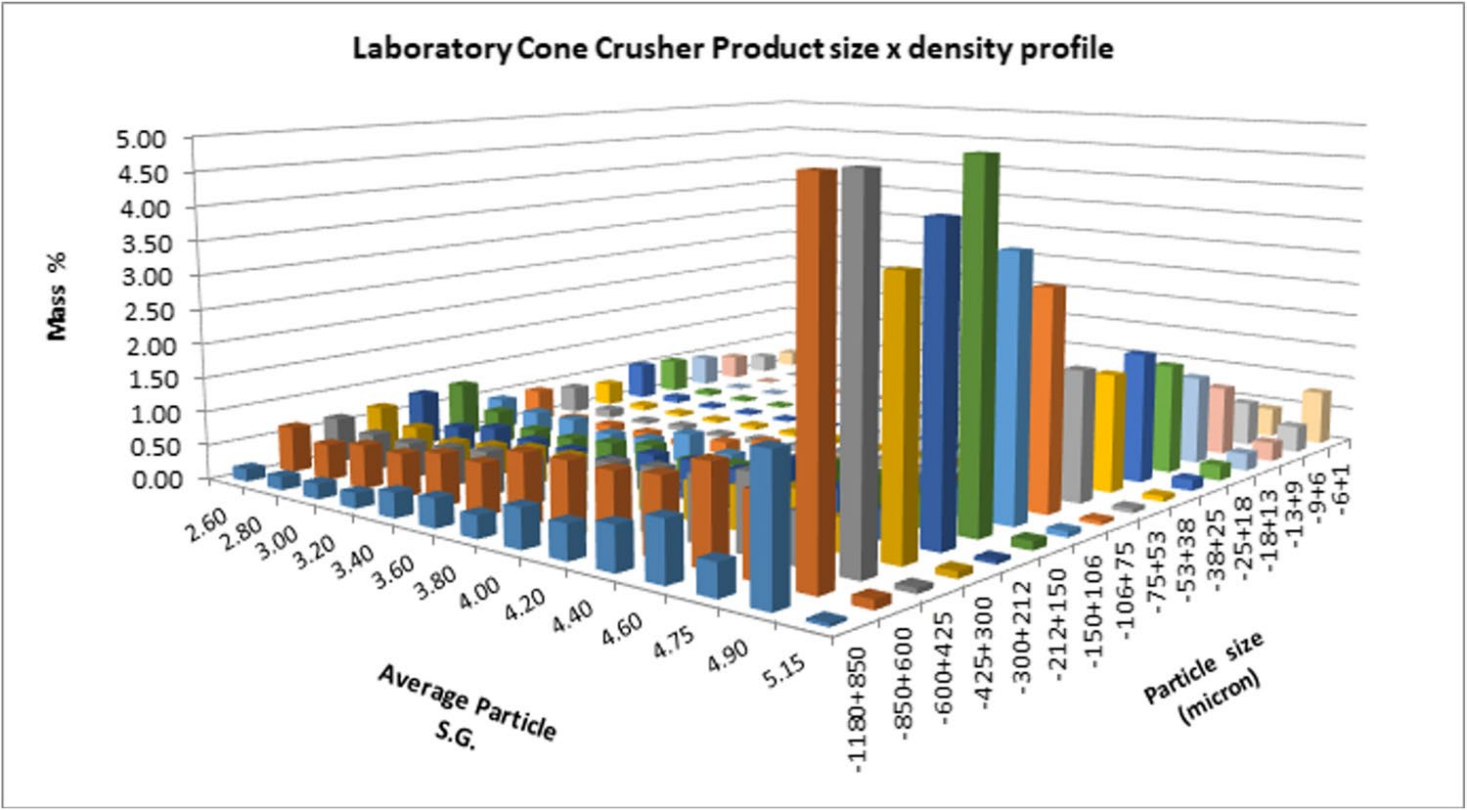
Feed size by density profile for the − 1180 µm crusher product

The density by size distribution of the − 1180 µm feed presented in Fig. 4 attempts to visually represent the contribution of each size class of the sample, highlighting the mass percent and amount of near-density fractions present. The presence of material in the intermediate densities of 3.40–4.75 for the coarser size fractions further demonstrates quartz dilution of the coarser particles.

### Shaking Table Test Work

Approximately 65 kg of the crusher product was wet screened at 212 µm utilising a Rhologan screen. A similar mass distribution was observed as per the size-by-assay results with approximately two-thirds of the sample reporting to the oversize with no preferential upgrade to either size class as indicated in Table 10.

**Table 10:** Feed wet screened at 212 µm

Size fraction (µm)	Mass (%)	Discrete grade (%)		Discrete deportment (%)	
		Fe	Si	Fe	Si
+ 212	60.9	44.1	16.5	60.6	61.8
− 212	39.1	44.7	15.9	39.4	38.2
Total (calculated)	100.0	44.3	16.3	100.0	100.0
Total (measured)		44.0	16.1		
% Relative error		0.7	1.0		

**Fig. 5:**
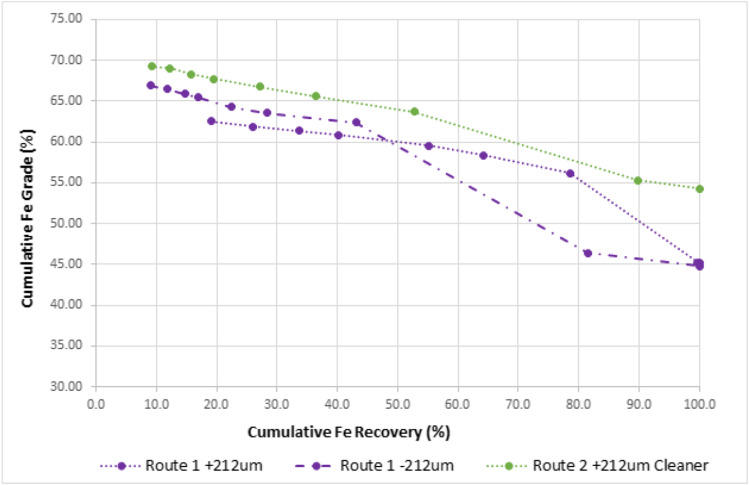
Cumulative Fe grade and recovery curve for the various size fractions for routes 1 and 2

The + 212 µm and − 212 µm size fractions from route 1 were independently processed on the shaking table and the unit cumulative Fe grade–recovery curve is presented in Fig. 5 (purple dotted lines). As expected, the − 212 µm fraction achieved higher product grades compared to the + 212 µm size fraction with the latter achieving a maximum product grade of 62.49% Fe at a unit recovery of 19.2%. At the same grade, the − 212 µm size fraction achieved over double the recovery (41.9%). The results correspond to the results of hematite liberation study and indicate that the + 212 µm size fraction will require milling to improve the liberation characteristics and thus product quality.

The + 212 µm concentrate from route 1 was milled to 80% − 212 µm and re-processed on the shaking table as a cleaner stage and is presented by the green dotted curve as route 2 in Fig. 5. The re-grind cleaner stage improved the product grade to a maximum of 67.72% at the same unit recovery of 19.2%. At unit recoveries below 40%, the cleaner stage has a similar profile to the natural − 212 µm fraction, although the latter achieved a better upgrade ratio (1.5 vs 1.3).

**Fig. 6:**
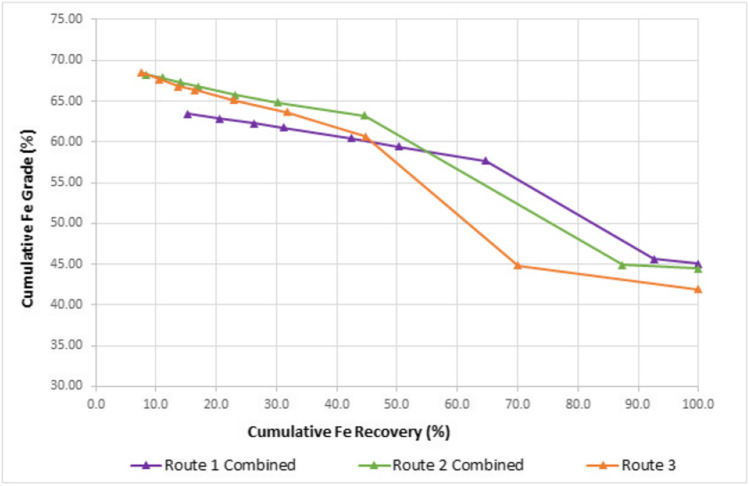
Overall cumulative Fe grade and recovery curve for routes 1 to 3

For comparative purposes, the shaking table results for the two size fractions were mathematically combined for the respective routes (1 and 2) and compared with route 3 in Fig. 6. Figure 6 clearly demonstrates that milling of the + 212 µm fraction (route 2 and 3) improves the overall product grades and recoveries that can be attained. However, at Fe recoveries exceeding 32%, route 2 achieves higher Fe recoveries than route 3. This could be attributed to 2.5 times more material reporting to the slimes product for route 3 than route 2. This indicates that pre-concentration prior to milling is critical to reduce fines generation and thus Fe losses. In addition, the reduced throughput to the mill (17.9% overall reduction for route 2) would lower CAPEX and OPEX costs as a smaller-sized mill would be required.

Vidyadhar et al. [10] investigated the beneficiation of BIF hematite from the Meghatuburu mine in eastern India. In their study, microscopic liberation analysis using the point count method (optical microscopy) was utilised to determine the hematite liberation grind. The results indicated that very fine grinding (− 150 μm) was required to liberate the gangue from the iron oxides. It is interesting that the liberation grind size is similar to the current study in which ~ 80% of the hematite reports to the − 212 µm size class. Based on the liberation study, Vidyadhar et al. [10] milled their sample grading 38.97% Fe to − 150 μm and subjected it to a gravity, magnetic separation and reverse flotation to achieve a product grading at 63.7% Fe at a yield and recovery of 28.0% and 45.8% respectively. This indicates that even at the liberation grind for BIF, under half the Fe recovery can be expected. However, since BIF is currently an unutilised available resource existing in large quantities any recovery at a reasonable product grade would improve the utilisation of this resource.

Table 11 compares the three options at selected Fe grades (60%, and 63% Fe). Of the three routes investigated, route 2 consistently provides better recoveries at each of the selected grades. At a product grade of 60% Fe, which would require blending with higher grade products, route 2 achieves a recovery of 52.1%. If a higher grade product of 63% Fe grade is targeted, a 7% Fe loss in recovery can be expected.

**Table 11:** Comparison of routes 1 to 3 at selected Fe grades

Route	Overall yield (%)	Fe grade (%)	Overall Fe recovery (%)
1	34.4	60.0	45.8
	13.7	63.0	19.1
2	41.0	60.0	52.1
	32.0	63.0	45.1
3	32.3	60.0	46.7
	23.0	63.0	34.5

However, by introducing magnetic separation on the tails and slimes, additional Fe could be recovered although this would increase CAPEX and OPEX costs.

## Conclusion

Mineralogical characterisation of the − 1180 µm crusher product grading at 44% Fe revealed that the dominant mixed hematite phase (phase C) is well liberated at 212 µm which is similar to a BIF hematite originating from India (39% Fe, liberation grind of 150 µm). Further work is recommended on similar BIF material in this grade range to determine if a pattern can be established.

Of the three gravity process routes investigated, the route that provided superior metallurgical results involved pre-concentration of the + 212 µm size fraction followed by re-grinding to − 212 µm and one final cleaning stage. The pre-concentration stage achieved 41.9% mass yield at Fe grade and recovery of 60.8% and 55.3%, respectively, while rejecting 83.3% of Si upfront. The advantages of this approach are twofold, firstly reducing slimes generation and thus Fe losses and secondly, 17.9% of the overall mass was rejected as waste and bypassed the mill reducing CAPEX and OPEX costs. This route (route 2) achieved Fe recoveries of 45.1 to 52.1% at Fe product grades of 63% and 60% respectively. By implementing a simpler gravity process, the South African BIF material was able to achieve similar Fe recoveries to that of the Indian BIF material at a product grade of 63.70% Fe. This study successfully demonstrates the beneficiation potential of the South African BIF material, a currently underutilised but dominant future resource.
